# Many faces of dominance: the manifestation of cohabiting companion dogs’ rank in competitive and non-competitive scenarios

**DOI:** 10.1007/s10071-024-01842-0

**Published:** 2024-03-02

**Authors:** Kata Vékony, Péter Pongrácz

**Affiliations:** Department of Ethology, ELTE Eötvös Loránd University, Pázmány Péter Sétány 1/c, Budapest, 1117 Hungary

**Keywords:** Companion dogs, Dominance hierarchy, Resource competition, Rank dynamics, Formal dominance, Agonistic behaviours

## Abstract

There are indications that companion dogs of multi-dog households form a hierarchy, maintained by formal and agonistic dominance. Although it was found that the behaviour of dogs depends on their rank in several contexts, so far, the assessment of their rank itself has been based on owner-completed questionnaires. With this research we endeavoured to find associations between rank scores from the Dog Rank Assessment Questionnaire (DRA-Q) and cohabiting dogs’ behaviour in a competitive test (Toy Possession test—32 dog pairs) and a non-competitive, citizen science scenario (Greeting test—20 dog pairs). Based on the grabbing the toy first and keeping the toy at the end variables, the dogs’ rank score provided a reliable indication of the dominant and subordinate dogs’ behaviour in the Toy Possession test. Similarly, the occurrence of dominant and submissive behaviours in the Greeting Test showed a good match with the agonistic and leadership subscores of the composite rank score from the DRA-Q. Our results provide a pioneering case for validating a questionnaire-based rank scoring method with biologically meaningful behavioural tests in the case of companion dogs. The finer analysis of the results highlighted that in the case of a multi-question scoring system, some components might provide more effective prediction of the dogs’ rank-related behaviour in some situations, while other components are more relevant in others, with traits related to agonistic dominance having relevance across contexts.

**Supplementary Information:**

The online version contains supplementary material available at 10.1007/s10071-024-01842-0.

## Introduction

Living in groups, either temporarily or permanently, is a common evolutionary phenomenon across the taxa of animals (Ward and Webster 2016). The benefits of group living include protection against predation, territorial defence, cooperative foraging and hunting, increased access to suitable mates and cooperative breeding (Creel and Macdonald 1995; Majolo et al. 2008). In the other hand, among the disadvantages of living in a group it is worth mentioning the easier spread of contagious diseases, attracting predators, and stronger competition for limited resources (Majolo et al. 2008). Animal groups can be very different regarding the complexity of social interactions and the sociability itself of the involved group members (Kutsukake 2009). While living in groups can facilitate the development of social bonds, conflicts may also arise around limited resources (Broom et al. 2009).

In groups of social animals, dominance-hierarchies form to minimize competitive conflict by regulating individuals’ access to these resources (Clutton-Brock and Huchard 2013). In the ethological sense, ‘dominance’ can be understood as a qualitative measure of a dyadic relationship based on the consistent outcomes of competitive interactions (Langbein and Puppe 2004).

While the development of dominance is based on agonistic contexts, in monkeys it was found that both intrinsic (age, social skills) and extrinsic factors (familiarity with the group, alliances with the others, preference by the females) also influenced the final rank and reproductive success of the individual males (Bernstein 1976; Bernstein and Gordon 1980). Moreover, the established rank can also be expressed outside of the actual competition, which is considered as an important factor of maintaining the acquired position in the hierarchy. Frans de Waal introduced the concept of formal dominance to describe rank-dependent affiliative displays and gestures between dominant and subordinate individuals (de Waal 1986).

Both formal and agonistic dominance have been described in wolves (Mech 1999; Schenkel 1967), with an emphasis on group cohesion rather than competition as being the ultimate ‘goal’ (Mech 1999; Packard 2003), with aggressive behaviours rarely occurring in wild-living wolf packs (Mech 1999), and having virtually no directional consistency in captive ones (van Hooff and Wensing 1987). Although dogs are the direct descendants of a wolf-like ancestor (e.g., Skoglund et al. 2015), the post-domestication social organization of dogs is shaped by very different ecological pressures: wolf packs are usually organized around family units (Mech and Boitani 2003), while free-ranging dogs (FRDs) are usually more loosely related to each other in a given group (Bekoff et al. 1984; Boitani et al. 2007; Boitani and Ciucci 1995). Wolves hunt in cooperation for large prey, FRDs are scavengers around human settlements (Boitani and Ciucci 1995; Majumder et al. 2014) and occasionally receive food directly from humans (Bhattacharjee et al. 2021). Wolves are cooperative breeders, and although some alloparental care have been described in dogs [free-ranging: (Paul et al. 2014; Paul and Bhadra 2018); companion: (Pongrácz and Sztruhala 2019)], the majority of the care falls on the mother, where she initiates the full weaning of the puppies from any sort of maternal resources much earlier—10–12 weeks of age (Boitani and Ciucci 1995; Paul et al. 2015; Paul and Bhadra 2017).

For the above reasons, it was thought for a long time that FRDs do not have strong group cohesion and do not form hierarchies (Boitani et al. 2007), but linear-like, age-graded hierarchies still have been described in their groups [Italy: (Cafazzo et al. 2010); India: (Pal et al. 1998)]. Measuring the hierarchy among them seems to be more reliable based on submission and formal displays, as the latter are more unidirectional, and the former having higher coverage than aggression in agonistic contexts (Cafazzo et al. 2010). Leadership in collective movement of FRD groups is not reserved for one dominant individual, and agonistic dominance alone does not predict the others’ following behaviour but receiving formal submissions in greeting and having more affiliative relationships (Bonanni et al. 2010a).

An earlier comparative study on captive wolves and similarly kept dogs (i.e., in a gamepark-like setting) found that dogs’ hierarchies tend to be less relaxed and tolerant than wolves’ (Range et al. 2015), but observations on FRD groups suggest that this might not be the case in a more natural setting (Bonanni et al. 2017).

The social lives of companion dogs (i.e., dogs that clearly have an owner), although they are conspecifics to FRDs, are shaped by very different environmental factors. FRDs routinely experience resource competition (Sarkar et al. 2019, 2023) and occasionally they show well-coordinated cooperation in intergroup conflicts such as territorial defence (Bonanni et al. 2010b). On the other hand, companion dogs’ access to resources and protection are fully controlled by the owner, so the necessity of a dominance-hierarchy to decrease intragroup competition and conflict is questionable (Bradshaw et al. 2009, 2016). Furthermore, the investigation of dominance-related behaviours in companion (or working) dogs is hampered by not only the ambiguous functional background for the mere need of such hierarchical structures, but understandably from ethical reasons, too. So far, empirical research is extremely scant on this topic and the existing research was mostly based on either questionnaire surveys or observational studies of more or less temporary dog groups.

Studies on dogs kept in kennels and enclosures found linear-like, tolerant hierarchies in dogs based on formal signals and agonistic behaviours (Range et al. 2015; Van Der Borg et al. 2015), with ambiguous relationships (Van Der Borg et al. 2015). The dogs in these studies were not FRDs, nor companion dogs in the strict sense, and their keeping environment vastly differed from that of owned dogs. But similar hierarchies were also found in groups of companion dogs in a doggy daycare (Trisko and Smuts 2015), although with less dominance displays within dyads and more friendly interactions (Trisko et al. 2016). Individuals in this study did not live together in the same households, so their social dynamics might not directly translate to groups of cohabiting family dogs that share the same house and same owners.

In turn, investigating hierarchies in cohabiting family dogs is usually done via questionnaire surveys assessing ranks of the individuals based on the owners’ description of their dogs’ interactions (Kubinyi and Wallis 2019; Pongrácz et al. 2008; Vékony et al. 2022), from the obvious reasons that instigating resource-competition situations among the dogs is risky and often would be considered as unethical. The surveys are sometimes accompanied, but not validated by behavioural observations (Ákos et al. 2014; Castro 2017). Many behavioural studies do not focus on how to measure rank, but accept the questionnaire-based assessment method as is, and investigate how the resulting rank associates with various traits and behaviours (Ákos et al. 2014; Lisberg and Snowdon 2009; Pongrácz et al. 2008, 2012; Vékony et al. 2022).

As currently there is no empirically validated hierarchy-questionnaire for companion dogs that live in multi-dog households, in this study we aimed to test the validity of our recently developed instrument (Vékony et al. 2022). We wanted to avoid the correlative approach (i.e., where dogs’ position in a hierarchy is compared to their behaviour in a non-related context, such as problem solving, (Pongrácz et al. 2008), thus we opted for setting up well-controllable competitive and non-competitive contexts that naturally happen in most households where at least two dogs are kept. As we tried capturing the possibly most natural scenarios, apart from an experimenter-directed situation (competition for different toys), we also opted for a citizen science solution. This latter solution was built around a short walk and reunion episode, where the owner takes only one of their dogs, leaving the other dog at home, then recording the greeting between the two dogs upon their reunion. Our goal was to find out whether the dogs’ behaviour during the direct resource competition (which one obtains the toy) and greeting behaviours upon reunion would show clear association with their rank score that was established via the questionnaire. We predicted that dogs in the dyads with higher rank scores will obtain the resource more in the resource competition scenario. We also expected to see the rank score mirrored in the dogs’ body postures during the greeting, and to see more dominant and less subordinate behaviours from dogs with higher scores.

## Materials and methods

### Questionnaire

For the assessment of the ranks of dogs we used our previously developed questionnaire (Vékony et al. 2022). Dog owners who had at least two cohabiting dogs in their households were recruited via advertisements placed on social media platforms. We did not offer any sort of incentive for their participation in the survey, and we used a convenience sample with snowball recruiting. Besides the demographic data of the dogs, the instrument consisted of eight questions about the behaviour of each dog in the household in everyday situations that are related to either formal or agonistic dominance. For each question, a dog could receive a score of 1 (response corresponds with dominant behaviour), − 1 (response corresponds with subordinate behaviour) or 0 (the owner indicated that their dogs exhibit the behaviour similarly depending on the context). Based on the average of the eight questions, each dog in each household obtained the mean Rank score between 1 and − 1. If the owner responded that a certain behaviour or situation never happens in case of their dogs, that question was excluded from the rank score calculation for those dogs (see the Rank Questionnaire in Supplementary Table S1). The questionnaire was completed for 1156 dogs from 518 households.

### Toy Possession test

#### Subjects

Subjects of this test were companion dogs older than 1 year old, living together, from such multi-dog households where their owners completed the previously mentioned questionnaire (*N* = 64, 32 dog pairs; *M*_age_ = 5.7, SD 3.07; *M*_agediff_ = 3.5, SD 2.6; 31 female, 25 neutered, 33 male, 26 neutered, from 18 different breeds and mongrels; 78% of owners were female, 22% male, *M*_age_ = 41.9, SD 10.3; 20 dog pairs came from 2-dog households, 6 from 3-dog households and 6 from more than 3-dog households). Members of each dyad lived together for at least 6 months before the test. Participation was voluntary and all owners signed an informed consent form. The experimenter explained to the owners the test protocol before the actual testing would start. The test could be interrupted or stopped by the owner or the experimenter if they noticed agonistic behaviour between the participating dogs. We were also prepared for that possible conflicts between the dogs were possible to be resolved with the help of a dog trainer on site (This never happened).

#### Experimental setup and equipment

The test was performed in fenced outdoor area (approximately 10 × 10 m) of a dog training school in Budapest. No training classes were held near the testing site while the tests were running.

The two reward types used in the test was a squeaky ball and a treat-dispensing dog toy.

**Fig. 1:**
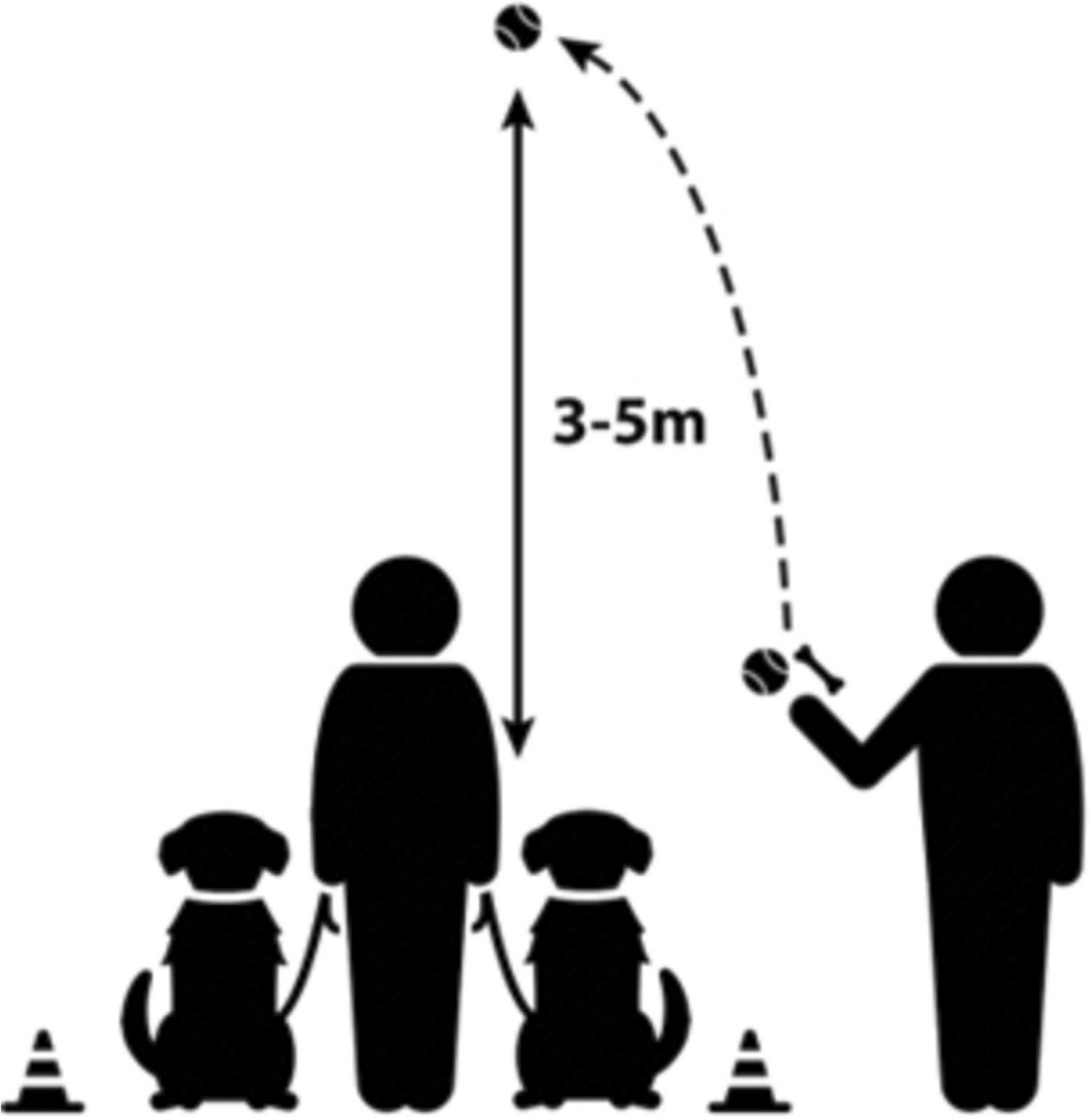
The experimental setup of the Toy Possession test

The starting line for the owner was marked with two cones used in dog sports. Figure 1 shows a schematic picture of the experimental setup.

The tests were recorded with two cameras (one regular Canon or Sony and a wide-angle GoPro action camera) that were positioned on tripods left and right from the starting point.

#### Testing procedure

This test was based on previous works done by Castro (2017) and Lisberg and Snowdon (2009). The test consisted of six identical trials, except the reward type used alternated between the trials. The owner stood with the two dogs on the starting line, holding them by the collar or harness. A female experimenter showed the reward to the dogs (stepped in front of them and squeaked the ball or let the dogs sniff the treat, put it in the toy and let them sniff the toy), then stepped in line with the owner, and tossed the toy approximately 3–5 m towards the midline, thus the ball fell the same distance from both dogs. It is important to note that while the experimenter tossed the ball (or toy), she avoided creating a typical ‘retrieval game scenario’, which means that the dogs were not given a priori verbal or behavioural cues that the ball (toy) will be tossed, and the dogs will need to retrieve (or get) it. In this way we tried to lessen the chance of heightened excitement in the dogs that could result is more fierce competition between them.

When the toy hit the ground, the owner let the two dogs go at the same time, using a release word (e.g., “go get it”). A trial lasted maximum 30 s, or until one of the dogs lost interest in the toy.

#### Exclusions

We had to exclude *N* = 2 dog pairs for the following reasons: in one pair one of the dogs was afraid when the experimenter approached the dogs with the squeaky toy; and in the case of the other pair of dogs the owner informed the experimenter that the dogs were trained to fetch in a fixed order (i.e., Dog B can never go for a ball or retrieval object before Dog A).

### Greeting test

#### Subjects

Subjects of this test were older than 1 year old companion dogs from multi-dog households (*N* = 38, tested in 20 dog pairs; *M*_age_ = 5.528, SD 3.14; *M*_agediff_ = 2.98; SD 2.36; 24 female, 21 neutered, 14 male, 10 neutered; all but one owner were female, *M*_age_ = 39.33, SD 12.1). Similarly to the subjects in the Toy Possession test, members of each dyad lived together for at least 6 months. Owners were recruited via Facebook and e-mail. An owner with more than 2 dogs could participate more than once, with different sets of two dogs at a time.

#### Experimental setup and equipment

As this was a citizen science project, owners performed the tests in their own homes, recording with their own cameras based on a written protocol and a video tutorial provided by us via e-mail. The requirement for the test site was that it had to be a room in the home of the participants, where one of their dogs can be left alone for a short period of time. The owners used two cameras (‘fixed’ and ‘mobile’) to record the test and uploaded the videos via Google Forms. Detailed description of the setup can be seen on the tutorial video in the Supplementary Material.

#### Testing procedure

The test was based on a situation where the owner takes one dog for a (short) walk while she/he leaves the other dog alone at home. We instructed the owners that apart of the video recording they should behave as naturally as they can during the test, thus likely capturing the usual behaviour of their dogs. The owner first had to set up the room and placed the fixed camera in a suitable position. At the beginning, the owner and both dogs were in the room. Then the owner prepared one of the dogs (Dog A) for a walk, started the recording on the fixed camera, and left the other dog (Dog B) in the room. After a short (approximately 10 min long) walk, they returned home, started the recording on the mobile camera and took off the leash Dog A before entering the room where Dog B was waiting.

When entering the room, the owner briefly greeted Dog B, but remained passive while recording the interactions of the two dogs for a minimum of 2 min. If the 2 min passed and the dogs did not interact for at least 30 s, or after maximum 5 min, the owner walked to the fixed camera with the mobile camera in their hand and used a sharp sound (e.g., whistle, click with the tongue) to provide a synchronizing cue for the two recordings before turning both cameras off. The protocol sent to the owners can be found in the Supplementary Material.

#### Exclusions

Not all owners sent in a 2-min video, but we did not exclude any videos that complied with the rest of the protocol and had at least 30 s footage recorded after the last physical interaction of the dogs. We only received one–one video of two dog pairs from the same owner, but we kept them in the analysis.

We had to exclude both videos of *N* = 2 dog pairs. Another person (aside of the owner) was present for both videos of one dog pair, and no sound was recorded for the other dog pair. We also had to exclude one of the videos of a dog pair, because no sound was recorded.

### Behavioural coding

The tests were recorded and coded with the Behavioural Observation Research Interactive Software (BORIS v. 7.13.6 © Olivier Friard and Marco Gamba (Friard and Gamba 2016)). Table 1 shows the behaviours coded in the Toy Possession test.

**Table 1:** Variables coded in the Toy Possession test

Variable name	Definition	Variable type
Start	1 if the dog starts to run towards the toy upon release, 0 if does not move or goes in another direction	Binary (1;0)
Grab	1 if the dog grabs the toy first in the trial	Binary (1;0)
Keep	1 if the dog has the toy in its mouth at the end of the trial	Binary (1;0)
Grab_time	Latency from release to the first grabbing of the toy	Continuous (s)

Each variable was coded for both dogs tested in the same pair

Behaviours coded in the Greeting test were based on (Trisko and Smuts 2015; Van Der Borg et al. 2015), focusing on body posture and interactions, with additional, owner-related behaviours (the coded body postures and most commonly occurring behaviours can be seen in Table 2, full ethogram is in Supplementary Table S2). We also grouped together the behaviours that show subordinance to create one binary variable and did the same with the typically dominance-related behaviours. 16% of the videos were coded by a blind coder who was unaware of the dogs’ Rank scores or the exact test protocol. For additional reliability, as the neutral body posture can vary between breeds and individuals, three experienced dog trainers also scored the average body posture of each dog in each video. While the original coding system differentiated between the dynamically changing posture of the dogs along the reunion episode, the task for the trainers was to provide a ‘summary score’ that characterized the body posture of the dog for the whole episode. Trainers’ scores ranged between 1 (lowest posture) to 5 (highest posture), with increments of 0.5. Score 3 represented ‘neutral’ for the given dog. Trainer 1 scored all the videos, trainer 2 scored all but two videos, and trainer 3 scored 2/3 of them.

**Table 2:** Most common variables coded in the Greeting test

Variable name	Definition	Variable type
pos_high	Tail: maximum highest carriage; ears: maximally erected (standing) or held forward (hanging)	Duration (s)
pos_halfhigh	Tail: partially highest carriage and held above the horizontal line of the back; ears: partly erected or hanging forward, higher than Neutral	Duration (s)
pos_neutral	Tail: follows line of hind quarter and held around the horizontal line of the back; ears: held relaxed, partly sideward	Duration (s)
pos_halflow	Tail: lower than Neutral but not held against or between the hind-legs; ears: partly retracted into the neck, lower than Neutral	Duration (s)
pos_low	Tail: the upper side of tail against hind quarter and S-shaped, or lower tucked between the hind-legs; ears: maximally retracted into the neck (standing) or held backwards (hanging)	Duration (s)
muzzlelick	The dog licks the other’s lips or chin	Duration (s) Frequency (1/s)
muzzle_bite	Inhibited biting over the other dog’s snout	Frequency (1/s)
bodytail_wag	Irregular movement of the tail with the hindquarter also moving	Duration (s)
chin_over	The dog places her head on the other’s back or shoulders	Duration (s) Frequency (1/s)
pass_under	Passing from the lateral side closely underneath the head of the other dog	Frequency (1/s)
interact	The dog initiates (and continues) close interaction with the other	Duration (s) Frequency (1/s)
owner_dur	The dog is looking at or orients towards the owner	Duration (s)

Each variable was coded for both dogs tested in the same pair

### Statistical analyses

We used R statistical software (v4.3.0, R Core Team, 2023) in Rstudio (Build 446, © Posit Software, PBC) with packages corrplot, DataExplorer, emmeans, fitdistrplus, glmmTMB, lme4, lmerTest, moments, MuMIn and outliers.

First, we calculated the Rank score for the participants of each test. Then we calculated subscores, namely “Formal” (only including licking the other dog’s mouth), “Agonistic” (including eating first or eating the other’s food, winning fights, obtaining valuable food items and better resting place) and “Leadership/defense” (including barking earlier/more at strangers, walking in front and defending the group) from subsets of the questionnaire.

We used Mixed Effects Logistic Regression (glmer with family ‘binomial’, with the pair ID and the dog ID as the random effects) to find if grabbing the toy and having the toy at the end of the trials associates with Rank score or any of the demographic factors. We used AIC based model selection to find the most parsimonious model.

For the Greeting test, we calculated a Body Posture Score for each dog in each trial based on the percentage of time they spent in high, low, or neutral body posture. Body Posture Score was calculated according to the following formula: BPS = (Pos_High% + Pos_Halfhigh%)*3 + Pos_Neut%*2 + (Pos_Low% + Pos_Halflow%)*1. For the calculation, we used the time percentages of different posture categories. We opted for pulling together the two categories originally belonging to the high and low posture, respectively because it turned to be difficult to differentiate between the fully and half-high postures. A Trainer Average score was also calculated from the three trainers’ scores for body posture. We used Linear Mixed Models to find if Rank score associates with body posture during the greeting. We used Beta Regression (glmmTMB with family ‘beta’, with the pair ID and the dog ID as the random effects) to find associations between interacting with the other dog (dependent variable) and Rank score, body posture, role in the test (staying at home vs. going for a walk) and demographic variables (independent variables). And finally, we used Mixed Effects Logistic Regression (glmer with family ‘binomial’, with the pair ID and the dog ID as the random effects) to find possible associations between exhibiting subordinate/dominant behaviours and Rank score.

## Results

### Toy Possession test

**Fig. 2:**
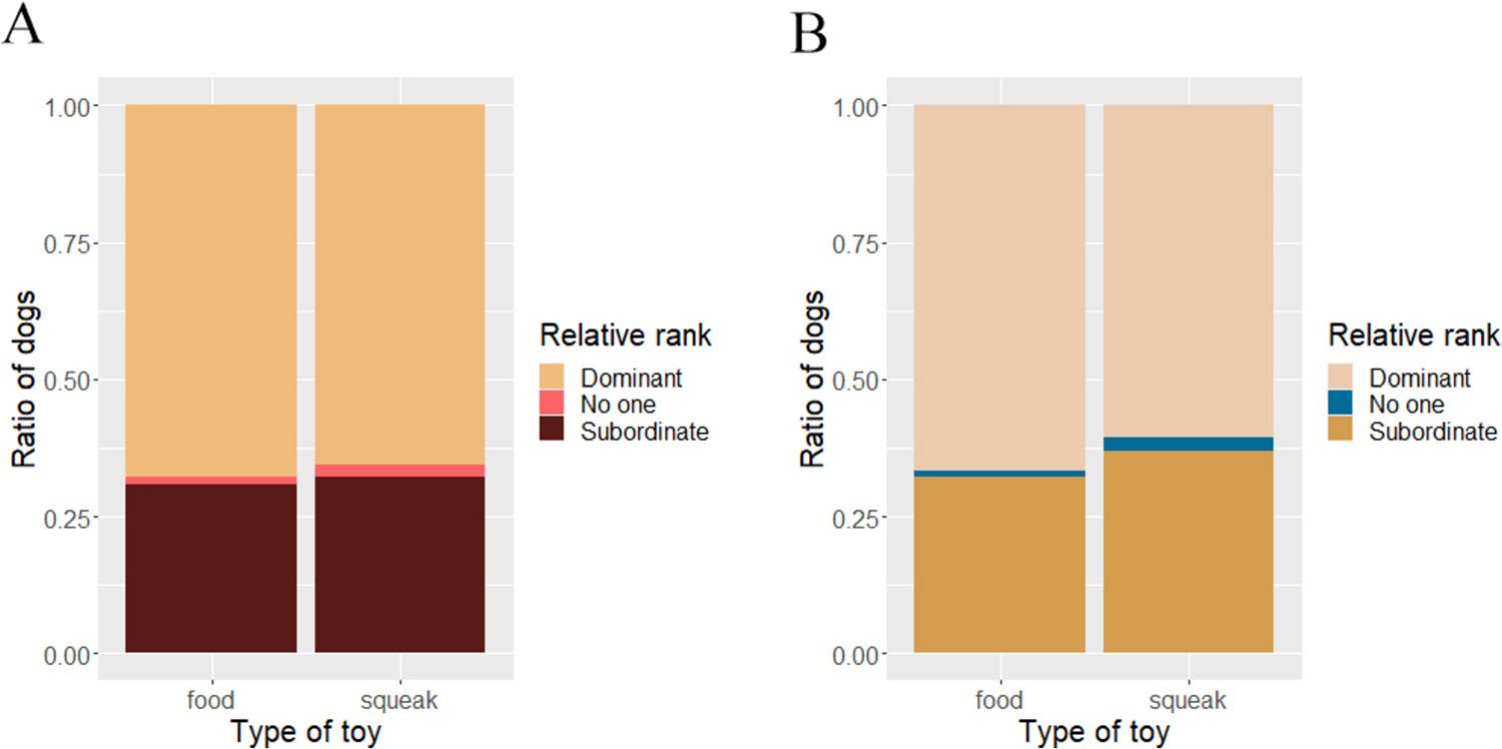
The association between relative rank of the dogs and grabbing the toy (**A**) and keeping (**B**) the toy at the end of the trial. In most of the trials, the dominant dogs grabbed (60%) the toy first and also kept it (57.2%), regardless of the toy type

Both dogs went out for the toy in 74% of the trials, but there was no trial when none of the dogs started to approach it. Dominant dogs grabbed and kept the toys most of the times, regardless of the type of toy. Dominant dogs grabbed the toy first in 60% of the trials, while subordinate dogs grabbed it first in 28.3%. No one grabbed the toy in only 3 trials of 2 dog-pairs together. Similarly, in 57.2% of the trials, the dominant dog had the toy at the end of the trials while this was 31% for subordinate dogs (Fig. 2). There were three dog pairs with the same Rank score for both dogs. In 55.5% of the trials, the older dog grabbed the toy first, and the younger one did it in 40%. In one dog-pair from the whole sample dogs were of the same age.

**Fig. 3:**
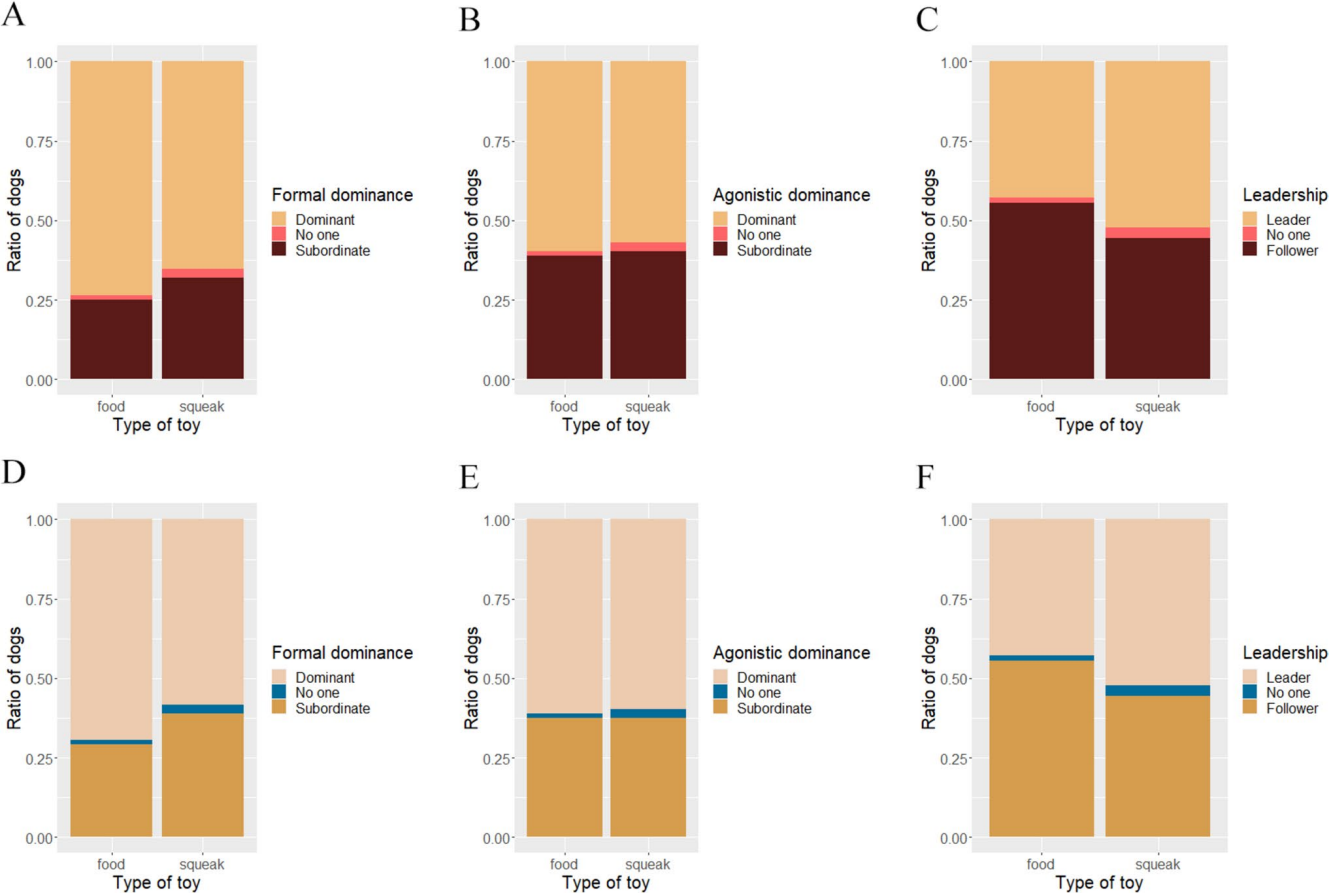
Dogs that receive more formal submission from their cohabiting conspecifics grabbed (**A**; *p* < 0.0001***) and kept (**D**; *p* < 0.0001***) the toy. Dogs that reportedly obtain more resources and win conflicts also grabbed (**B**; *p* = 0.0129*) and kept (**E**; *p* = 0.0125*) the toy more often, but leading and defending the group did not have this association (**C**, **F**; *p* = 0.97; *p* = 0.8)

When examining Formal rank, Agonistic rank, and Leadership rank (determined by the subscores), formally dominant dogs grabbed the toy in 55.56% of the cases, and they kept it in 51.11%, compared to the 22.78% and 27.22% when formally subordinate dogs grabbed and kept it. Six dog pairs had the same formal ranks. The ratio was similar for the agonistic rank, dominant dogs grabbed the toy in 46.67% of the trials versus the 31.67% when subordinate dogs grabbed it, and dominant dogs had the toy at the end of 48.33% of the trials and subordinate dogs had in only 30%. Six dog pairs had the same Agonistic scores. In the case of Leadership rank, 9 dogs had the same score, thus the same rank, and the ratio of dominant versus subordinate dogs grabbing and keeping the toy was more balanced: dominant dogs grabbed the toy in 35% and subordinate dogs grabbed it in 33.33% while dominant dogs had the toy by the end of the trial in 33.33% of all trials and subordinate dogs had it in 35% (Fig. 3).

We found significant association between Rank score and grabbing the toy [*β* = 2.09, SE 0.0282, *z* = 3.426, 95% CI (0.541–3.586), *p* = 0.0133], but no other variable (age, sex, reproductive status, number of dogs in the household) was included in the model. In case of keeping the toy, the model also only included the Rank score, but here the association was only a trend [*β* = 1.264, SE 0.7206, *z* = 1.754, 95% CI (− 0.2305 to 2.8253), *p* = 0.079].

When using the subscores separately, we found a significant association between Agonistic rank score and grabbing the toy [*β* = 1.622, SE 0.8028, *z* = 2.02, 95% CI (− 0.2449 to 3.3965), *p* = 0.0434]. As Formal rank also had a significant association with grabbing the toy: Tukey post-hoc tests showed that subordinate dogs grabbed the toy significantly less than dominant dogs [*β* = − 3.21, SE 0.915, *z* = − 3.507, 95% CI (− 5.35 to − 1.064), *p* = 0.0013]. This model had a better fit than using the Rank score (∆AIC = 6.52; *p* = 0.0053).

Agonistic rank score also had a significant association with keeping the toy [*β* = 1.778, SE = 0.7768, *z* = 2.290, 95% CI (0.0594–3.2861), *p* = 0.022], while Leadership score showed a nonsignificant trend [*β* = − 1.3066, SE = 0.7486, *z* = − 1.745, 95% CI (− 2.947 to 0.2973), *p* = 0.08]. Formal rank also had a significant association: Tukey post-hoc test showed that subordinate dogs were less likely to keep the toy than dominant ones [*β* = − 2.139, SE = 0.807, *z* = − 2.651, 95% CI (− 4.03 to − 0.248), *p* = 0.0219]. This model fit better than the one using Rank score (∆AIC = 8.84; *p* = 0.0018). We found no other significant associations (neither the age and sex nor reproductive status had an effect).

### Greeting

Of the 14 different rank related behaviour variables coded, only the following occurred at least in one of the videos: muzzlelick (7 videos), bodytail_wag (7 videos), pass_under (5 videos), chin_over (6 videos), muzzle_bite (1 video), growl (1 video), and with one exception in the muzzlelick and one in the muzzle_bite, the behaviours occurred only once or twice per video. The dogs spent on average 30% of the recorded time looking at the owner and only 13.9% in close physical interaction with each other. The time spent with interaction was relatively short, except for one dog pair that spent 66% of the time playing in one video and 57% on the other. Four videos showed no interaction between the dogs.

The body posture scores given by the three trainers had moderate to strong correlations (Tr1–Tr2: *r* = 0.49, *p* < 0.0001; Tr1–Tr3 *r* = 0.46, *p* = 0018; Tr2–Tr3 *r* = 0.726, *p* < 0.0001), but only Trainer 1’s score had a weak correlation with the coded Body Posture Score (*r* = 0.295, *p* = 0.016), neither the other trainer’s scores, nor the three trainers’ average score did not correlate with it. Trainer 3’s score had a weak negative correlation with the coded time spent in low body posture (*r* = − 0.31, *p* = 0.04) and Trainer 1’s score had a weak positive correlation with time spent in neutral body posture (*r* = 0.31, *p* = 0.01) and a weak negative association with half low body posture (*r* = − 0.36, *p* = 0.003). The average score by trainers had weak correlations with these three postures as well (*r*_low_ = − 0.24, *p*_low_ = 0.045; *r*_halflow_ = − 0.33, *p*_halflow_ = 0.006; *r*_neutral_ = 0.26, *p*_neutral_ = 0.036). In the subset coded by the blind coder, there was a strong correlation in the time spent in high body posture (*r* = 0.778, *p* = 0.0029), but not other postures and nor the Body Posture Score.

This deemed the Body Posture Score based on the coding unreliable. We used the Trainer Average score for further analyses.

**Fig. 4:**
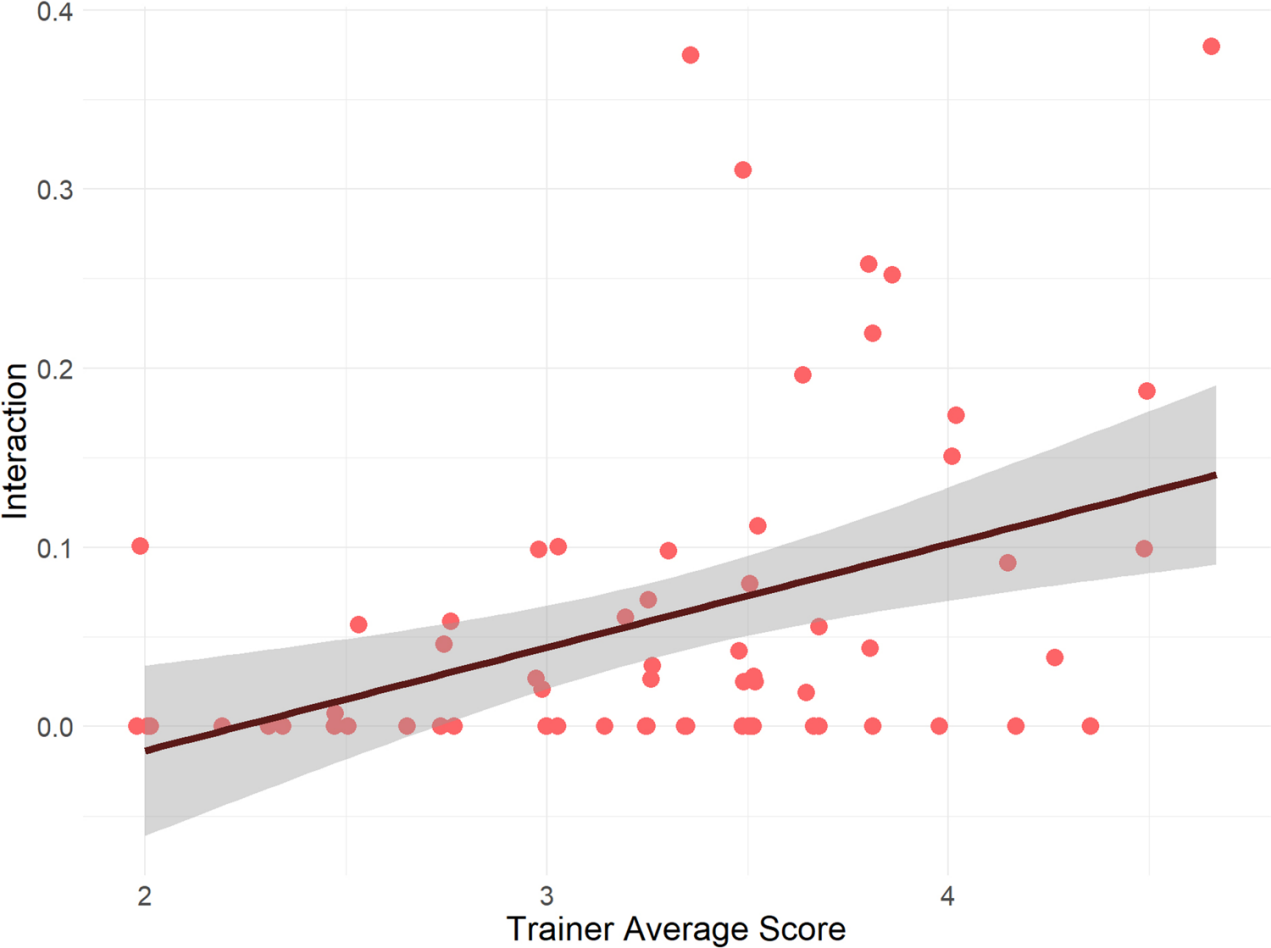
Dogs that received higher posture scores from the Trainers initiated more interactions and spent more time with trying to get into interaction with the other dog (*p* = 0.03811*)

**Fig. 5:**
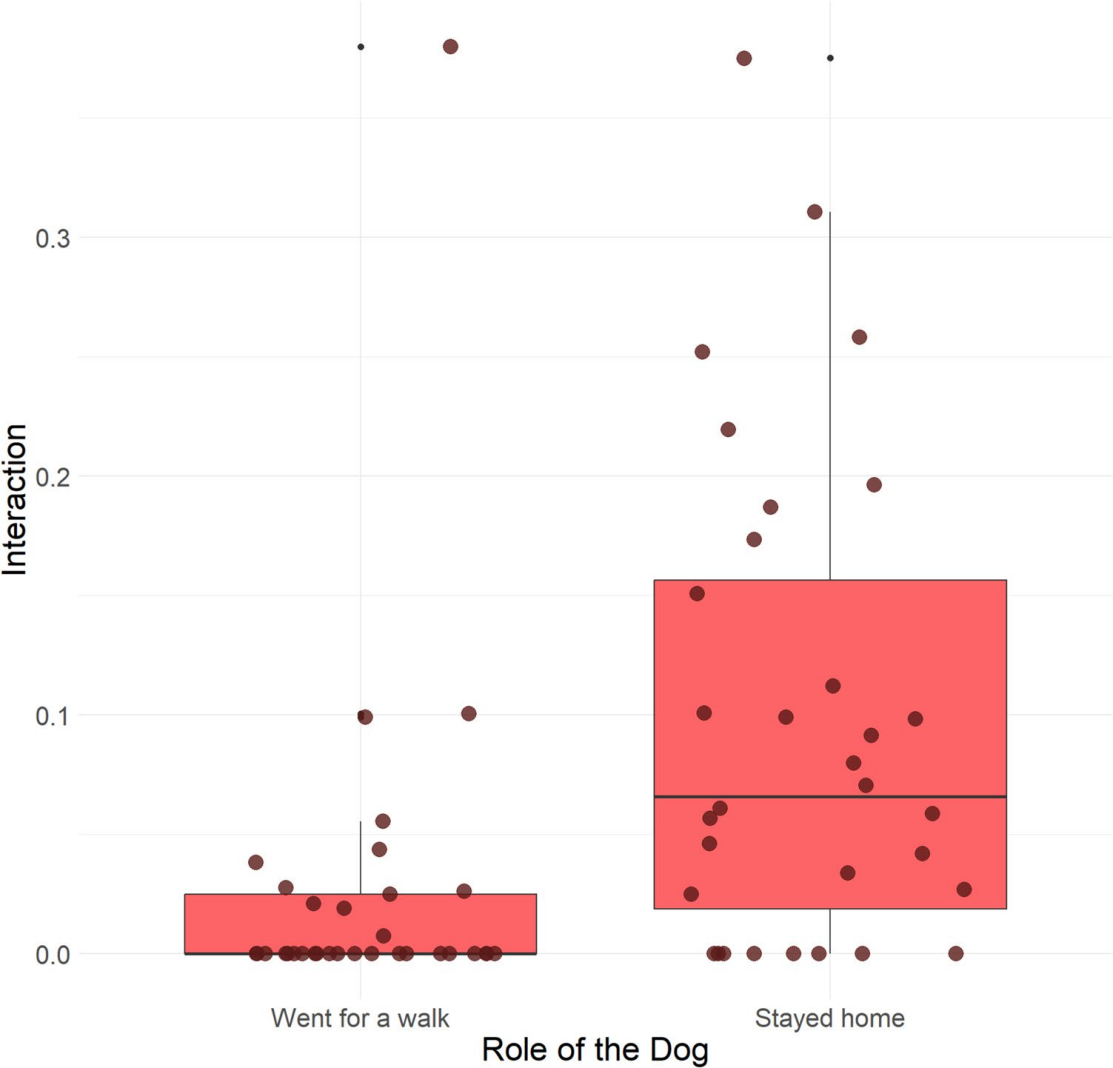
Dogs that stayed home initiated more of the interactions than the dogs that went for a walk with their owner (*p* = 0.0019**)

The Trainer Average score had a significant positive association with initiating and maintaining interactions with the other dog [*β* = 0.4688, SE 0.2261, *z* = 2.074, 95% CI (0.0257–0.9118), *p* = 0.03811; Fig. 4]. The only other variable that affected interaction was the dog’s role in the test: post-hoc test showed that the dogs that stayed home initiated more interactions with the arriving dog than vice versa [*β* = − 0.904, SE 0.291, *z* = − 3.11, 95% CI (− 1.47 to − 0.334), *p* = 0.0019 Fig. 5].

**Fig. 6:**
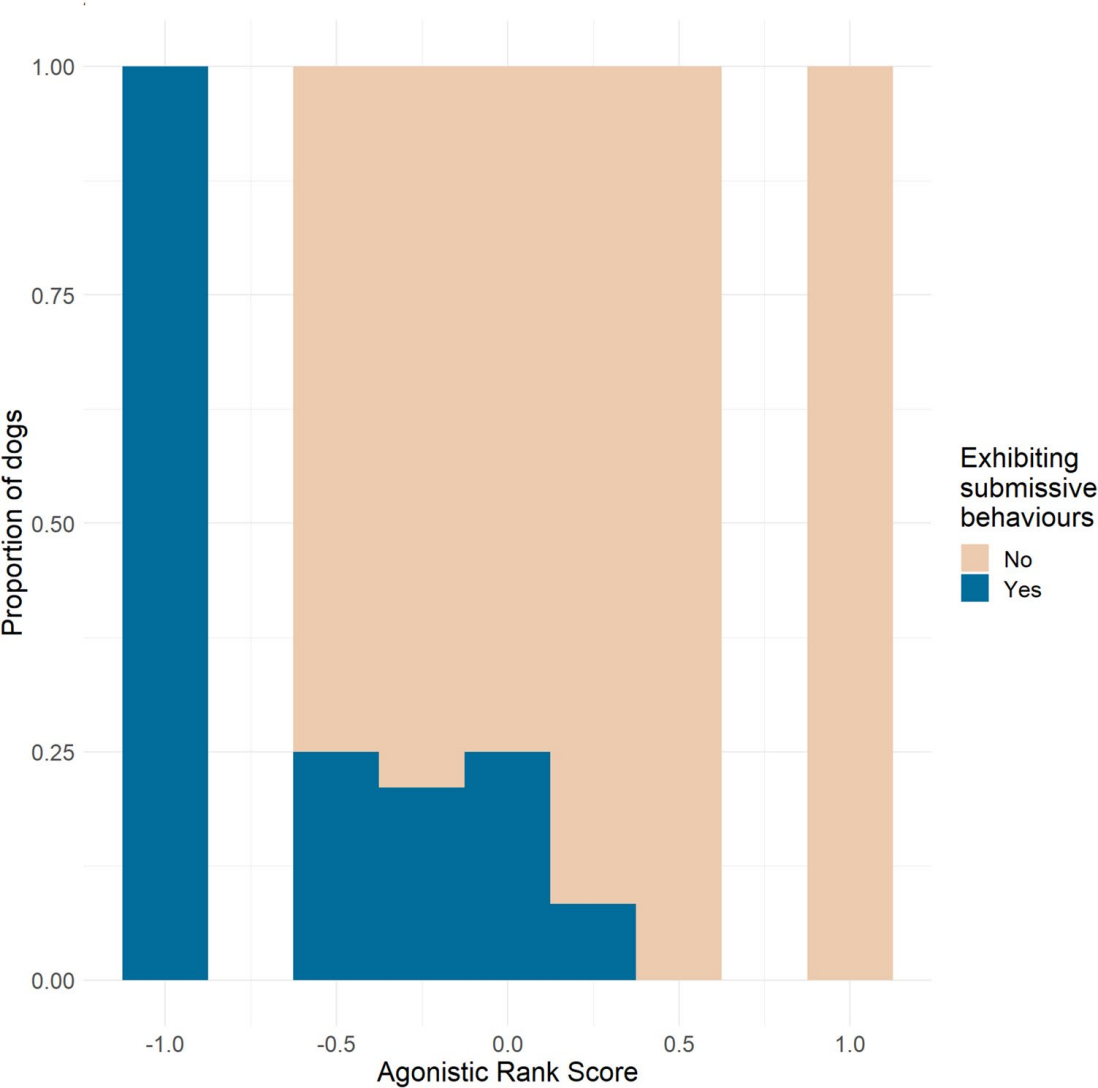
Proportion of dogs displaying submissive behaviours during the greeting. Agonistic Rank Score had a significant association with submissive behaviours (*p* = 0.0129*)

Rank score had a non-significant negative trend with exhibiting subordinate behaviours [*β* = − 1.574, SE 0.838, *z* = − 1.878, 95% CI (− 3.9804 to − 0.153), *p* = 0.06], and no association with dominant behaviours. With the subscores separately, we found a significant negative association between Agonistic rank score and subordinate behaviours [*β* = − 2.6361, SE 1.06, *z* = − 2.486, 95% CI (− 5.9623 to − 0.7466), *p* = 0.0129; Fig. 6]. This model fit better than the one using Rank score (∆AIC = 1.29; *p* = 0.0056). Agonistic rank score also had a significant positive association with exhibiting dominant behaviours [*β* = 2.797, SE 1.417, *z* = 1.974, 95% CI (0.2328–6.763), *p* = 0.048], while they had a nonsignificant negative trend with the Leadership score [*β* = − 1.909, SE = 1, *z* = − 1.909, 95% CI (− 5.0612 to − 0.1293), *p* = 0.056]. None of the other factors (age, sex, reproductive status, number of dogs in the household or being the staying or the walking dog in the test) had any effect.

## Discussion

In this complex study, we evaluated the rank-relationship of cohabiting companion dogs with the help of the owners who completed the Dog Rank Assessment Questionnaire [DRA-Q, developed by Vékony et al. (2022)]. With two additional tests, the resource competition-based Toy Possession test and the non-competitive Greeting test we could successfully validate the Rank Scores that were provided by the questionnaire. Specifically, we found that dogs with higher rank scores obtained and kept the toy more frequently, and this result was not confounded by the age of the dogs. Remarkably, the same associations were found between obtaining and keeping the toy and several (but not all) items of the DRA-Q—namely dogs that receive submissive displays from the other dog, win conflicts and obtain more often the resources according to the owners’ observations, prevailed in the Toy Possession test, too. In the Greeting test, we also found that those items of the DRA-Q that describe agonistic behaviours within the dog dyads, had reliable and biologically meaningful associations with both the dominant and submissive behavioural displays between the reuniting dogs, while behaviours corresponding to leadership show trend-like association with dominant displays. Thus, based on these results, our questionnaire is the first empirically validated instrument for the assessment of cohabiting companion dogs’ rank-relationships.

Although ‘dominance’ (both in the meaning of formal and agonistic displays) is a widely (and unfortunately, most of the time incorrectly) used concept in popular science and applied fields as dog training (Bradshaw et al. 2009; Herron et al. 2009), the associations between dogs’ ranks and particular behaviours were actually rarely investigated. In spite of the promising findings between their rank and social learning performance (Pongrácz et al. 2008, 2012); leadership during dog walks (Ákos et al. 2014); or dogs’ personality (Vékony et al. 2022) so far all of these studies relied on the assessment of dogs’ rank via various questionnaires, where the answers provided by the owners have never been validated against such behaviours that could be connected to the formation or maintenance of hierarchical relationships between cohabiting dogs. Thus, although such a validation was clearly warranted (Kubinyi and Wallis 2019), so far no attempts were made to test questionnaire-based rank scores against dominance-related behaviours in companion dogs.

Our main aim was to provide empirical and quantitative evidence for not only the existence, but the measurability and generalizability of hierarchies in groups of cohabiting companion dogs. We also aimed to behaviourally validate the Dog Rank Assessment Questionnaire (Vékony et al. 2022), as although the questions used for assessment all possess ethological validity (Kubinyi and Wallis 2019), their real-life applicability was never investigated empirically. The contribution of the dog owners always comes with the possibility of observer and/or response bias in case of questionnaires [e.g., Essner et al. 2020)]. Furthermore, not all behaviours described in the questionnaire even occur in all companion dog groups and excluding them from the assessment might skew the results. On the other hand, different items in the questionnaire correspond to different aspects of rank and different ‘types’ of dominance, which are although not independent from each other and usually overlap, not necessarily coincide completely (Bonanni et al. 2010a). As our subjects were companion dogs, it is possible that particular owners actively try to prevent agonistic behaviours between their dogs (Mehrkam and Wynne 2021), or some of our subjects simply cannot express group-defending, or leadership behaviour because of the lack of suitable scenarios in their lives. For this reason, we did not only calculate dogs’ Rank scores and assessed relative ranks, but broke the complex Rank score down further to separately investigate Formal rank, Agonistic rank and Leadership.

In our sample we found dyads of dogs with same Rank Score values. This ambiguity may show that owners are not always aware of the competitive situations between their dogs, but it also can be the indicator of an ‘egalitarian’ scenario in particular households. According to Hand (1986), when the resource has similar value for the group members, or subjective resource values are highly variable but the cost of potential conflict is symmetrical, egalitarianism can become an adaptive system against risky outcomes of agonistic interactions.

The results supported our prediction that dogs’ relative ranks assessed with the DRA-Q reliably translate to biologically valid behaviours in both competitive and non-competitive contexts. This result is especially important because it shows that (1) the hierarchy of companion dogs indeed manifests itself in resource competition, even if dog owners usually try to minimize the chance for such scenarios; and (2) it fits well to those earlier results that found associations between dogs’ rank and non-competitive sociocognitive capacities such as learning from a demonstrator in a problem-solving task (Pongrácz et al. 2008, 2012). Our finding that higher ranked dogs showed less submissive and more dominant behaviours towards the other dog—and vice versa—during the greeting test, independently of their actual role (stay home or return from a walk) underlines the stability of dogs’ rank position in multi-dog households. One could predict that in such asymmetric contexts, when one dog clearly takes the favourable position for a while (being on a walk alone with the owner) the other dog would try to re-establish its position after the reunion. Indeed, investigation on jealous-like behaviour in dog-dyads showed rank-independent jealousy in dogs when the owners turned their attention towards another dog (Abdai et al. 2018). However, in our test, the temporary separation of the two cohabiting dogs uniformly prompted dogs’ motivation to express their ranks upon reunion with their companions, especially in the form of submissive displays exhibited by lower ranking dogs.

The only aspect where being the dog who went for a walk or stayed at home had a significant effect with the behaviours after the reunion was that the dogs who stayed at home initiated more interactions with the other dog after its return. Curiously, no such difference was found in the owner-directed behaviours. Still, we can speculate that the asymmetry between the dog-directed interaction initiations of the participating dogs might be connected to the difference they just experienced in their closeness to their owner. Dog owners play a central organizing role in companion dogs’ life [attachment: (Topál et al. 1998); jealousy: (Abdai et al. 2018); main ‘limited resource’: (Vékony et al. 2022)], and the way how the Greeting Test was orchestrated induced a temporary asymmetry in the two dogs’ exclusive access to the owner. Although this did not affect the influence of rank on the behaviour of the reuniting dogs’ behaviour, the dog who had the chance to have an exclusive time with the owner during the walk ignored its partner more likely after arriving home again. This result is in agreement with those reports that highlighted the lessened cooperativity with conspecifics and elevated willingness to cooperate with humans in group-living dogs (Range et al. 2019), because one can assume that the dog who had the chance for one-by-one interaction with the owner would subsequently pay less attention to the other dog.

While a study that previously used the Toy Possession Test for rank assessment found that age plays a bigger role in acquiring both types of toys than rank (Castro 2017), we found no such effect. Dominant dogs grabbed and kept the toys significantly more often than subordinate ones, regardless of whether the toy was filled with food or if it was a squeaky ball. This corresponds with rank’s most fundamental role and meaning, namely the preferably aggression-free access to the bigger share or first access in case of resource competition (Drews 1993). It is important to remember that in our Toy Possession Test we never noticed aggressive interactions during the dogs’ attempts to get to the toy first. As the participants were cohabiting companion dogs, presumably the well-established rank relationship between them made it possible that the outcome of the ‘resource competition’ for the toy was usually a clear-cut issue.

During the Toy Possession test, we did not encounter aggressive interactions between the members of dog dyads. Although grabbing and keeping of the toy was less frequently performed by the lower ranking dogs, in such cases more dominant individuals did not intervene. This can be explained by the ‘dominant respect’ phenomenon (Kummer and Cords 1991), when the dominant animal does not attempt forcefully obtaining a resource that has been already claimed by a subordinate individual.

In our interpretation, Rank score could provide a more detailed information on dog groups’ social dynamics than the binary dominant/subordinate classification of two individuals’ relative ranks, as it quantifies this qualitative measure, and comparing Rank scores of all individuals within the group instead of just dyads separately could inform on the ‘steepness’ of the hierarchy, not just on a dyadic, but on a group level as well. This property of Rank score is reflected in the results, as it had a positive association with grabbing the toy in the Toy Possession Test. But not all aspects or types of dominance contributed to this: we found that formal dominance [receiving formal/affiliative submissions (Cafazzo et al. 2010)] and agonistic dominance (winning fights and obtaining valuable resources) associates with both acquiring and keeping the toys, but leadership does not. This detail warrants for the potential reconsideration of the predictability of some of the questionnaire items in different contexts. While the questions regarding the formal and agonistic rank performed effectively in our validating attempts with a competitive behavioural test, the leadership-related questions failed to do so, while the question regarding formal dominance had no relevance in the non-competitive test. The uneven efficacy of the individual questionnaire items can also explain why Rank score as a whole was less effective than the more detailed subscore approach in our tests. The binary dominant/subordinate classification in a dog dyad is based on the sum of individual answers given to each dog’s questions. When a question is not applicable to some dyads, the rank sum will not reflect that particular behaviour in their case, degrading the ‘resolution’ of the Rank score (but not necessarily affecting the final rank). Thus, if Rank score is based on less predictive questions, its predictability will be less powerful to the behaviour of dogs in a given experimental context.

Taking both tests into account, Leadership score was the least relevant, having no effect in the competitive test, and only having a weak trend in the non-competitive test. This finding is in parallel with earlier research on mammalian hierarchies that found no correlation between group defence or leadership and rank (Rowell 1974). This is further supported by Bonanni and colleagues’ study on free ranging dogs, as they found that dogs receiving more submissions from the others, which is a classical indicator of social rank, did not take more risks or engage more in intergroup conflicts (Bonanni et al. 2010b), although they took the group-leading role more often (Bonanni et al. 2010a).

In our earlier, questionnaire study (Vékony et al. 2022) we found indirect evidence that in dogs ‘dominance’ is not an independent trait of the animal’s personality, contrary to some interpretations in human psychology (e.g., Wilks 2009). However, it seems like that the cohabiting dogs’ rank shows clear associations with most of the personality traits from the Big Five model, and this effect was mainly independent from the effect of age. The causality of this association is unknown and most probably it is a complex interrelationship between rank dynamics and the various personality components, however, it all fits to the ethological concept of rank. According to this, the rank position of an individual dog would depend on its ability to consistently ‘win’ competitive interactions ‘against’ the other dog. Once the hierarchy was formed, active (agonistic) competition will occur less frequently and intensely. However, as our results in the behaviour tests showed, the correlates of the hierarchy (i.e., having higher or lower rank) persistently manifest themselves in such scenarios where the two dogs interact—either in mildly competitive (Toy Possession test) or non-competitive (Greeting test) situations. The common feature of these events was the lack of open aggression and the clear outcome that was well-predictable based on the rank score gained by the DRA-Q instrument as a whole, or on its subscores related to agonistic and formal dominance and leadership, depending on the test situation.

A limitation of this study is that although we recruited participants from households where various numbers of dogs live together from two to five dogs, dogs participated in the tests as dyads, so more complex group dynamics were not possible to observe directly. A limitation specific to the Toy Possession test only is the question of subjective resource value (Bradshaw et al. 2009) of the toys to the individual dogs, regardless of their overall rank. Despite our best efforts, some dogs also might have interpreted the test as a play scenario. While playfulness is suggested to have an association with rank (Anestis 2005), a study on companion dogs found no association between playing fetch and retrieving the ball and dogs’ rank (Kubinyi and Wallis 2019). Although the results of the present study are convincing, because we used two somewhat similar object as resources, the generalizability remains still somewhat limited.

The Greeting Test also comes with its own difficulties. The dogs’ behaviour in the test might be affected by factors not just out of our control, but knowledge as well. For example, whether the owner regularly takes the dogs out for walks separately, or just the opposite, the situation is completely novel to them. The owner’s actions during the test might be confusing to the dogs, especially if they have a fixed routine for daily walks or an established greeting ceremony with the owner. These unknown factors might give some explanation for the failure of the body posture coding, as well as the fact that studies on both free-ranging and companion dogs have found that agonistic dominance displays such as high posture has lower coverage and directional consistency than submissive displays (Cafazzo et al. 2010; Trisko and Smuts 2015). The other problem with body posture coding in our study was the wide variety of appearance, morphology, and anatomy of the dogs in our sample, and also the single observation event. Van Der Borg and colleagues successfully used body posture as an indicator of rank in their study, but all dogs in their sample were purebreds with known ‘neutral’ ear and tail positions, and they observed the dogs for 12 weeks, which might be enough time for the investigators to become more sensitive to subtle individual differences (Van Der Borg et al. 2015).

A limitation in case of both tests was that we could not investigate if the duration of how long the dogs were living together would influence their behaviour during the tests. Time spent together might affect social behaviour directly, but this effect also might be mediated through the hierarchy which also takes time to establish. Our subjects were adult dogs living together for at least 6 months, which could be enough to establish hierarchy, but we did not ask the owners about possible recent changes in their dog group, such as adopting a new dog or death of an older dog that can also induce changes in the hierarchical structure among the cohabiting dogs.

Although both tests come with limitations and difficulties, together they provide strong evidence on the validity of the DRA-Q as a reliable instrument to assess rank related social dynamics in groups of cohabiting companion dogs. Our results also provide a firm basis for using questionnaire-based rank assessments for companion dogs in the past and future investigations, but also warrant for a careful inclusion of questions regarding the various forms and contexts where rank-dependent interactions might occur.

## Supplementary Information

Below is the link to the electronic supplementary material.

Supplementary file1 (XLSX 95 kb)

Supplementary file2 (DOCX 26 kb)

## Data Availability

Raw data, including anonymous demographic data of participants, are supplemented in the electronic Supplementary Material.
