# Supplementary material for: Many faces of dominance: the manifestation of cohabiting companion dogs’ rank in competitive and non-competitive scenarios
Source: Anim Cogn. 2024 Mar 2;27(1):12. doi: 10.1007/s10071-024-01842-0 (PMC10907432; doi:10.1007/s10071-024-01842-0)
Supplement: Supplementary file 2 — Supplementary file2 (DOCX 26 kb) [file 10071_2024_1842_MOESM2_ESM.docx]

Supplementary Material

Supplementary material for manuscript “*Many faces of dominance – the manifestation of cohabiting companion dogs’ rank in competitive and non-competitive scenarios*” submitted to **Animal Cognition**.
Authors: Vékony, Kata*^1^ & Pongrácz, Péter^1^Author affiliations: ^1^ Department of Ethology, ELTE Eötvös Loránd University, Budapest, Hungary
Corresponding author: Kata Vékony (kata.vekony.kami@gmail.com)

**Table S1.** The rank questionnaire from Vékony et al. 2022. Rank score is calculated as an average of the questions. NA answers do not count towards the Rank score. The letter after each question indicates to which subscore it belongs (F: formal, A: agonistic, L: leadership)

|  | This dog | One of my other dogs | Depends on the situation/Similarly | Not applicable |
| --- | --- | --- | --- | --- |
| When a stranger comes to the house, which dog starts to bark first (or if they start to bark together, which dog barks more or longer)? (L) | 1 | -1 | 0 | NA |
| Which dog licks more often the other dog's mouth? (F) | -1 | 1 | 0 | NA |
| If the dogs get food at the same time and at the same spot, which dog starts to eat first or eats the other dog's food? (A) | 1 | -1 | 0 | NA |
| If the dogs starts to fight, which dog usually wins? (A) | 1 | -1 | 0 | NA |
| If they got a special reward (e.g., a marrow bone), which dog obtains it? (A) | 1 | -1 | 0 | NA |
| Which dog goes in the front during walks? (L) | 1 | -1 | 0 | NA |
| Which dog acquires the better resting place? (A) | 1 | -1 | 0 | NA |
| If your dogs are being attacked, which dog faces the threat in the front? (L) | 1 | -1 | 0 | NA |

**S1.** Instructions for the Greeting test

*Both the instructions and the video subtitles were originally in Hungarian and translated only for the purpose of attaching them to the manuscript.*

Dear Owner,

Thank you for participating in our Citizen Science project. Please carefully read the instructions and follow the steps. You can also find the link to a tutorial video at the bottom of the page.

Description of the Greeting tests:

In this test, first you’ll take **one of your dogs** for a short, **10-minute walk**, then **record the greeting** between the two dogs, and later **repeat the test with the dogs’ roles reversed**. For this test, you will need **two video recording devices** (smartphone, camera, laptop webcam, etc…). One should be in a **fixed place** from which it can record the **place where the dogs will meet** after the walk. The **recording** should be started on this camera **before leaving** with the dog, so it won’t miss the beginning of the greeting between the dogs. You should **take the other camera** with you and start the **recording right before entering** the room. **Record at least 2 minutes**, but if the dogs continue interacting, continue recording, but not for more than **5 minutes**.

Exact steps:

1. Set the *fixed* camera to cover as much of the room as possible, especially the door and its surroundings, and the part of the room where the dogs will most likely interact.
2. Start the recording on this camera.
3. Take the *mobile* camera with you.
4. Prepare one of the dogs (*Arriving dog*) for a walk as usual and step out the door. Leave the other dog (*Staying dog*) in the room. All doors of the room should be closed, so the dogs won’t move out of the cameras’ frames after returning.
5. Upon returning, but before entering the room, start the recording on the *mobile* camera, and remove the leash from the *arriving dog*, so both dogs can move freely.
6. Enter with the dog. If the *staying dog* wants to greet you, you can greet and pet them briefly, but remain as passive as possible.
7. If possible, always hold the *mobile* camera in a way both dogs are in frame. You can move and step away from the door to achieve this.

If the two dogs lose interest in each other and move to separate places, hold the *mobile* camera according to the following:

1. IF only one dog is in the frame of the *fixed* camera, record the other dog with the *mobile* camera.
2. IF none of the dogs in is the frame of the *fixed* camera, keep the *mobile* camera on the *staying dog*.
3. IF both dogs are in the frame of the *fixed* camera, but you can only record one with the *mobile* camera, keep the *mobile* camera on the dog whose body posture, movements and behaviour is likely to be less visible on the *fixed* camera (the dog is turned away from the *fixed* camera, or farther away from it, etc…). If you can’t decide, keep the *mobile* camera on the *staying dog*.
4. The recording should be no less than 2 minutes. If the 2 minutes passed, and the dogs did not interact with each other for at least 30 seconds, you can stop the recording. If they still interact, continue recording up to 5 minutes maximum.
5. Before stopping the recording on the cameras, bring the *mobile* camera near the *fixed* one, and use a short, sharp sound (clicker, whistle, etc…) to provide a synchronizing cue for the two recordings. Make sure both cameras record both the audio and the source of the sound.

Important details:

- Make sure the *fixed* camera has enough storage space for both the duration of the walk (approximately 10 minutes) and the greeting (2-5 minutes), so it won’t stop recording prematurely;
- Both cameras should be able to record audio;
- No other person or dog should be in the room during the walk or the greeting;
- If only one dog is visible, do not switch the *mobile* camera back and forth between the dogs (you can switch between the dogs if this way both will be visible on at least one of the recordings, e.g.: the dog you’ve been recording moves in the frame of the *fixed* camera, so you switch to the other – out of frame – one);
- If only one dog can be visible (both are out of the *fixed* camera, and only one fits in the frame of the *mobile*), it should be the *staying dog*;
- Do not have food or toys in the room;
- The dogs should not be able to leave the room.

Repeat the test with the dogs’ roles reversed on another day. Try to make the second test in a similar time of the day to the first one. If it’s not possible to record on another day, make sure to have at least 8 hours between the tests.

If you have more than 2 dogs, you can do the test multiple times in different pairings, but make sure to have 2 videos of each pair with the roles reversed.

Please rename the videos in the following way: [Staying dog’s name]_fix and [Staying dog’s name]_mobil. E.g.: Buddy_fix and Buddy_mobil.

Link to the tutorial video: XXX

If you have any questions, please contact us at XXX

You can upload the videos on this form: XXX

**S2. Video s**link: <https://youtu.be/wINN4-s4p2I>

**Table S2.** Variables coded in the Greeting Test. Body Posture definitions are not fully suitable for all dog breeds and mongrels that participated. Duration percentages calculated from not the whole test duration, but the duration the dog was visible.

| **Behavior code** | **Description** | **Behavior type** | **From** |
| --- | --- | --- | --- |
| pos_high | Tail: maximum highest carriage; Ears: maximally erected (standing) or held forward (hanging). | Duration (s) Duration % | van Der Borg et al. 2015. |
| pos_halfhigh | Tail: partially highest carriage and held above the horizontal line of the back; Ears: partly erected or hanging forward, higher than Neutral. | Duration (s) Duration % | van Der Borg et al. 2015. |
| pos_neutral | Tail: follows line of hind quarter and held around the horizontal line of the back; Ears: held relaxed, partly sideward. | Duration (s) Duration % | van Der Borg et al. 2015. |
| pos_halflow | Tail: lower than Neutral but not held against or between the hind-legs; Ears: partly retracted into the neck, lower than Neutral. | Duration (s) Duration % | van Der Borg et al. 2015. |
| pos_low | Tail: the upper side of tail against hind quarter and S-shaped, or lower tugged between the hind-legs; Ears: maximally retracted into the neck (standing) or held backwards (hanging). | Duration (s) Duration % | van Der Borg et al. 2015. |
| muzzlelick_freq | The dog licks the other’s lips or chin. | Frequency (1/s) Duration (s) Duration % | Trisko & Smuts 2015. |
| muzzlelick_dur |  |  |  |
| bodytail_dur | Irregular movement of the tail with the hindquarter also moving. Cannot be paired with higher than neutral posture. | Duration (s) Duration % | van Der Borg et al. 2015. |
| passunder_freq | Passing from the lateral side closely underneath the head of the other dog. Cannot be paired with higher than neutral posture. | Frequency (1/s) | van Der Borg et al. 2015. |
| stare_dur | Intense fixating look with tensed body, for at least 2 seconds. Cannot be paired with lower than neutral posture. | Duration (s) Duration % | van Der Borg et al. 2015. |
| growl_dur | Low-pitched rumbling vocalization. | Duration (s) Duration % | van Der Borg et al. 2015. |
| teeth_freq | Baring of the teeth. | Frequency (1/s) Duration (s) Duration % | van Der Borg et al. 2015. |
| teeth_dur |  |  |  |
| snap_freq | Biting while moving one step towards the other dog, without physical contact. | Frequency (1/s) | van Der Borg et al. 2015. |
| bite_freq | Biting any part of the other dog with pressure that could cause harm. | Frequency (1/s) | van Der Borg et al. 2015. |
| shrink_freq | Quick movement away from the other dog (1-2 steps). | Frequency (1/s) | van Der Borg et al. 2015. |
| flee_freq | Running away from the other dog while turning away from them. | Frequency (1/s) | van Der Borg et al. 2015. |
| chin_over_freq | The dog places her head on the other’s back or shoulders. | Frequency (1/s) Duration (s) Duration % | Trisko & Smuts 2015. |
| chin_over_dur |  |  |  |
| muzzle_bite_freq | Inhibited biting over the other dog’s snout. | Frequency (1/s) | Trisko & Smuts 2015.. |
| bark_freq | Barking at the other dog. | Frequency (1/s) | van Der Borg et al. 2015. |
| tail_wag_dur | Regular sideways tailwag. | Duration (s) Duration % | van Der Borg et al. 2015. |
| paw_on_dur | Placing one or both front paws on the other dog's head or back. | Duration (s) Duration % | van Der Borg et al. 2015. |
| interact_freq | Initiating interaction with the other. | Frequency (1/s) | own |
| interact_dur | Interacting with physical contact (coded for the initiating dog). | Duration (s) Duration % | own |
| own_dur | Looking at, orienting towards or interacting with the owner. | Duration (s) Duration % | own |
| shake_freq | Shaking the body. | Frequency (1/s) | own |
| dog_not_visible | The dog is not visible on either camera’s recording. Cannot be paired with posture or behaviour. | Duration (s) Duration % | technical |
| Start-Stop | The test ends if 30 seconds passed without interaction. |  | technical |
